# Femoral revision with the direct anterior approach

**DOI:** 10.1007/s00064-022-00768-5

**Published:** 2022-05-31

**Authors:** Martin Thaler, Kristoff Corten, Michael Nogler, Boris Michael Holzapfel, Joseph Moskal

**Affiliations:** 1 Arthroplasty Center, Helios Klinikum Munich West, Steinerweg 5, 81241 Munich, Germany; 2 Department of Orthopedics and Traumatology -Experimental Orthopedics, Medical University Innsbruck, Innsbruck, Austria; 3 Center of Orthopaedics, Trauma Surgery and Rehabilitation Medicine, University of Greifswald, Greifswald, Germany; 4 Hip Unit, Orthopedic Department, Heuppraktijk, Ziekenhuis Oost-Limburg Genk, Herselt, Belgium; 5 Department of Orthopedics and Trauma Surgery, Musculoskeletal University Center Munich (MUM), University Hospital, LMU Munich, Munich, Germany; 6 Department of Orthopedic Surgery, Virginia Tech Carilion School of Medicine, Institute for Orthopedics and Neurosciences, 24014 Roanoke, VA USA

**Keywords:** Direct anterior approach, Revision total hip arthroplasty, Femoral revision, Approach extensions, Modular stem, Direkter vorderer Zugang, Revisionsendoprothetik, Femorale Revision, Erweiterungen des Zugangs, Modularer Schaft

## Abstract

**Objective:**

The advantages of the direct anterior approach (DAA) in primary total hip arthroplasty as a minimally invasive, muscle-sparing, internervous approach are reported by many authors. Therefore, the DAA has become increasingly popular for primary total hip arthroplasty (THA) in recent years, and the number of surgeons using the DAA is steadily increasing. Thus, the question arises whether femoral revisions are possible through the same interval.

**Indications:**

Aseptic, septic femoral implant loosening, malalignment, periprosthetic joint infection or periprosthetic femoral fracture.

**Contraindications:**

A draining sinus from another approach.

**Surgical technique:**

The incision for the primary DAA can be extended distally and proximally. If necessary, two releases can be performed to allow better exposure of the proximal femur. The DAA interval can be extended to the level of the anterior superior iliac spine (ASIS) in order to perform a tensor release. If needed, a release of the external rotators can be performed in addition. If a component cannot be explanted endofemorally, and a Wagner transfemoral osteotomy or an extended trochanteric osteotomy has to be performed, the skin incision needs to be extended distally to maintain access to the femoral diaphysis.

**Postoperative management:**

Depending on the indication for the femoral revision, ranging from partial weight bearing in cases of periprosthetic fractures to full weight bearing in cases of aseptic loosening.

**Results:**

In all, 50 femoral revisions with a mean age of 65.7 years and a mean follow-up of 2.1 years were investigated. The femoral revision was endofemoral in 41 cases, while a transfemoral approach with a lazy‑S extension was performed in 9 patients. The overall complication rate was 12% (6 complications); 3 patients or 6% of the included patients required reoperations. None of the implanted stems showed a varus or valgus position. There were no cases of mechanical loosening, stem fracture or subsidence. Median WOMAC (Western Ontario and McMaster Universities Osteoarthritis Index) score before surgery improved significantly from preoperative (52.5) to postoperative (27.2).

## Abstract (de)

**Operationsziel:**

Die Vorteile des direkten vorderen Zugangs zur Hüfte („direct anterior approach“, DAA) bei der primären totalen Hüftendoprothetik als minimal-invasiver, muskelschonender und internervaler Zugang werden von zahlreichen Autoren beschrieben. Daher wurde der DAA für primäre Hüftendoprothesen in den letzten Jahren zunehmend beliebter, und immer mehr Ärzte entscheiden sich für diesen Zugang. Somit stellt sich die Frage, ob femorale Revisionen durch dasselbe Operationsintervall durchgeführt werden können.

**Indikationen:**

Aseptische oder septische Femurimplantatlockerung, Fehlstellung, periprothetische Infektion oder periprothetische Femurfraktur.

**Kontraindikationen:**

Fistel von einem anderen Zugang.

**Operationstechnik:**

Die Inzision des primären DAA kann distal und proximal erweitert werden. Falls notwendig, können zwei Muskelablösungen erfolgen, um das proximale Femur besser freizulegen. Das DAA-Intervall kann bis zur Spina iliaca anterior superior erweitert werden, um den Tensor abzulösen. Falls notwendig, können die Außenrotatoren zusätzlich abgelöst werden. Falls eine Komponente endofemoral nicht explantiert werden kann und eine transfemorale Osteotomie nach Wagner oder eine erweiterte Trochanterosteotomie durchgeführt werden muss, sollte die Hautinzision distal erweitert werden, um den Zugang zum Femurschaft aufrechtzuerhalten.

**Weiterbehandlung:**

Das postoperative Management ist abhängig von der Indikation für die Femurrevision und reicht von Teilbelastung bei periprothetischen Frakturen bis zur Vollbelastung bei aseptischen Lockerungen.

**Ergebnisse:**

Insgesamt wurden 50 Femurrevisionen mit einem durchschnittlichen Alter 65,7 Jahren und einem durchschnittlichen Nachuntersuchungszeitraum von 2,1 Jahren untersucht. Eine endofemorale Revision konnte in 41 Fällen durchgeführt werden, während ein transfemoraler Zugang mit Lazy-S-Extension bei 9 Patienten durchgeführt wurde. Die Komplikationsrate betrug 12 % (6 Komplikationen). Drei Patienten benötigten eine Revisionsoperation. Keiner der implantierten Schäfte zeigte eine varische oder valgische Positionierung. Ebenso zeigte keiner der implantierten Schäfte eine mechanische Lockerung, Schaftfraktur oder eine Sinterung. Der mediane WOMAC(Western Ontario and McMaster Universities Osteoarthritis Index)-Score verbesserte sich im präoperativen (52,5) zu postoperativen Vergleich (27,2) signifikant.

## Introductory remarks

Over the past 20 years, the direct anterior approach (DAA) has been popularized for total hip arthroplasty (THA) in a minimally invasive fashion [11]. Nowadays, the indications for primary THA have been extended for primary THA in more complex cases and/or younger patients [5], and that consequently extended the indications for revision THA. Projections expect that the rate of primary and revision THA will increase significantly over the next 20 years [4]. In the initial period of the DAA for THA it was assumed that the anterior approach can only be used for primary THA. Femoral revisions can be performed with different surgical approaches to the hip joint. The posterior, the anterolateral and the direct lateral approach are the most commonly used approaches for revision total hip arthroplasty. However, recent publications have shown that the DAA can also be used for many types of revision surgeries [1–3, 9]. Recent cadaver reports have shown that femoral revision arthroplasty can theoretically be safely performed through the DAA interval [1, 6]. Other reports have proven that the DAA can be successfully used for the treatment of periprosthetic femoral fractures with the extension of the DAA [8] or for two-stage septic revision arthroplasty [10]. All published data show good results after revision THA performed with the DAA. Stem revision through the DAA interval for aseptic loosening showed similar results compared with other surgical approaches in terms of complications, clinical outcome, and dislocation rate. These results indicate that femoral revision with the DAA interval is a safe and reliable procedure. Therefore, DAA can be used safely as a standard operative approach for all kinds of THA revisions. For femoral revisions, the incision can be extended distally and proximally to provide better exposure of the entire femur.

However, adequate training and experience are needed to perform revisions through the DAA [7]. Special instruments and anatomic knowledge are mandatory for the success of these procedures. This article summarizes the currently available surgical techniques and results to perform femoral revisions through the DAA interval.

## Surgical principle and objective

Femoral revisions can be performed with different surgical approaches to the hip joint. The posterior, the anterolateral and the direct lateral approach are the most commonly used approaches for revision total hip arthroplasty. However, the interval of the direct anterior approach can be safely used for all indications of femoral revision arthroplasty. The femur can either be approached directly with the primary DAA interval (endofemoral approach) or with approach extensions (approach of the femoral diaphysis). Two possible releases can be performed in order to optimize femoral exposure during surgery.

Surgeons who have been trained in the DAA can also perform revisions through the same interval. In the revision setting the primary incision can be used or extended.

## Advantages

- No muscular transection is needed in easy cases (exceptions: tensor release, distal or proximal extension of approach)
- Damage to periarticular soft-tissues can be minimized in some cases
- Primary skin incision can be used
- Easy orientation, for surgeons familiar with the DAA
- DAA-trained surgeons can use their favorite approach for revisions
- Supine positioning of the patient
- Easy intraoperative determination of leg length
- Easy application of intraoperative fluoroscopic control
- Good visualization of the anterior acetabulum if needed

## Disadvantages

- Technically demanding procedure
- Posterior exposure of acetabulum is limited
- Potential complaints associated with lateral femoral cutaneous nerve lesions: meralgia, hypesthesia

## Indications

- Aseptic loosening of cemented and uncemented stems
- Periprosthetic fracture
- Periprosthetic joint infection
- Malalignment of the stem
- Leg length discrepancy
- Impingement

## Contraindications

- A draining sinus from another approach
- The need to remove a posterior acetabular plate in the same surgery
- The need to remove a custom-made implant, which was implanted through different approach

## Patients information

- General surgical risks, e.g., thrombosis, infection, wound healing problems, postoperative hemorrhage
- Potential complications of revision surgery (infection, dislocation, neurological complaints, loosening, fracture, etc.)
- Potential numbness or a burning sensation in the anterolateral region of the thigh and, in the worst cases, dysesthesia or meralgia due to injury to the LFCN (lateral femoral cutaneous nerve)

## Preoperative work up

- Templating of the femoral implant
- Select size, length and off-set of revision implant
- Preoperative aspiration of the hip joint and 14-day incubation of the culture to exclude infection as a possible cause of loosening of the prosthesis
- In case of suspected periprosthetic joint infection: joint aspiration, laboratory tests, clinical findings
- In aseptic cases: single shot intravenous antibiotic administration
- Intravenous administration of tranexamic acid, if not contraindicated
- Preparation of a cell saver. Autotransfusion
- Clipping of hair of the leg from the belly button to below the knee joint

## Instruments and implants

- Curved Hohmann retractors (standard minimally invasive THA Hohmann retractors)
- Double-tipped Hohmann for femoral elevation
- Instruments for stem and if needed cement removal
- Standard revision stems
- In cases of periprosthetic joint infection: femoral spacer
- Cerclage wires, cables or plate in cases of periprosthetic fractures
- Traction table if surgeon also performs primary DAA with a traction table

## Anesthesia and positioning

**Fig. 1:**
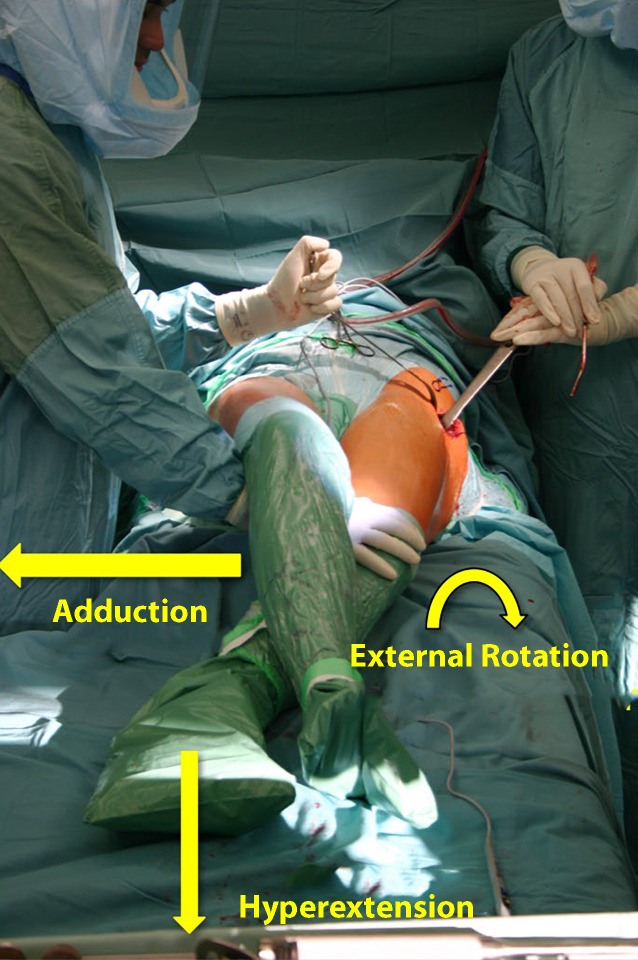
The patient is positioned on a standard operating room table or an extensions table in supine position. A table attachment opposite to the operated side (e.g., arm-board) allows for easier hyperabduction of the opposite leg during femoral exposure. The extension table is not mandatory for revisions with the direct anterior approach (DAA). Both legs are draped flexibly (only the operative leg needs to be sterilely draped, but sterile draping of both legs may be helpful for the surgical exposure). During femoral revisions, a combination of adduction, hyperextension and external rotation of the operated leg is needed to guarantee adequate femoral exposure. The operated leg is placed beneath the contralateral leg. (left hip)

- General or spinal anesthesia depending on the length of estimated surgical time
- Supine position
- Antibiotic prophylaxis
- Free draping of operated limb in order to enable proper manipulation during exposure of the femoral shaft
- External rotation, adduction, and hyperextension of the operated leg should be provided throughout the entire surgery to guarantee good exposure for the femur
- Operated leg must be placed underneath the contralateral leg in order to have good exposure to the femur (Fig. 1)

## Surgical technique

**(**Fig. 2, 3, 4, 5, 6, 7, 8, 9, 10, 11, 12, 13, 14)

**Fig. 2:**
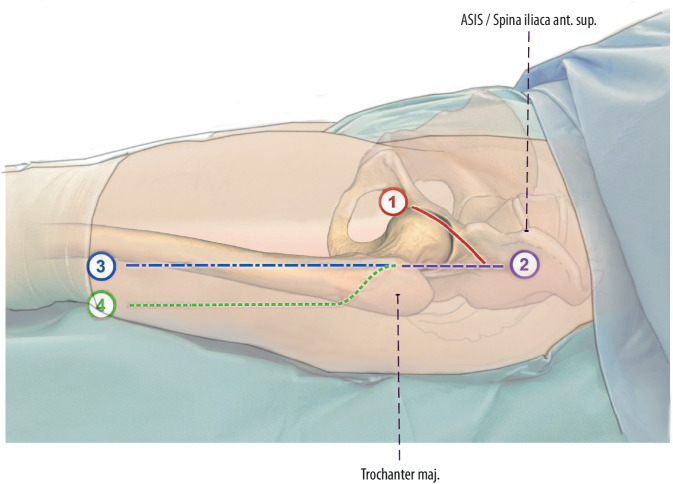
All possible skin incisions defined as a direct anterior approach (DAA) and its extensions include (1) skin crease bikini incision, (2) longitudinal skin incision, (3) longitudinal extension, and (4) lazy-S extension (right limb). Proximally, the skin incision can be extended to the anterior superior iliac spine (*ASIS*). The skin crease bikini incision is recommended exclusively for primary total hip arthroplasty (THA), cup revisions, and easy endofemoral revisions. For complex femoral revisions, a longitudinal skin incision is recommended. (left hip)

**Fig. 3:**
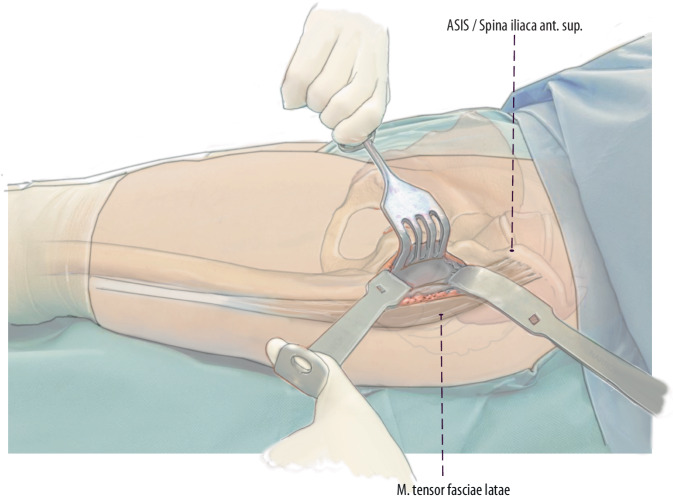
The first step is to perform a primary longitudinal skin incision with the proximal starting point two finger breadths lateral and two finger breadths distal to the ASIS. Like in primary THA, the fascia of the tensor fasciae latae (TFL) is incised, and the TFL is mobilized laterally to expose the interval. If not approached during the index surgery, ascending branches of the lateral circumflex vessels need to be identified and cauterized. After incising the deep layer of the tensor fascia, the retractors can be placed around the hip joint. After an anterior capsulectomy, the hip can be dislocated anteriorly and the femur can be approached in the interval. (left hip)

**Fig. 4:**
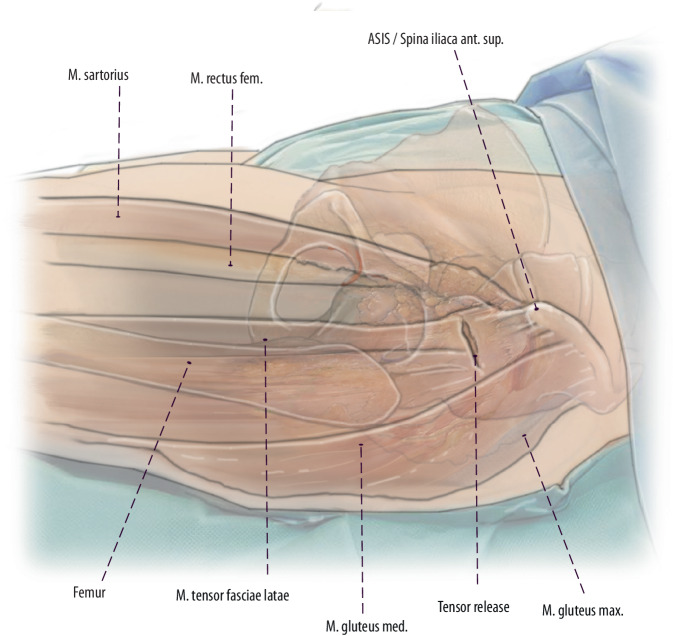
After proximal extension to the level of the ASIS, about one third of the TFL muscle can be released 1–2 cm distally to its origin at the ASIS. This TFL release is situated within the tendinous structure´s of the tensor muscle and facilitates simple refixation. (left hip)

**Fig. 5:**
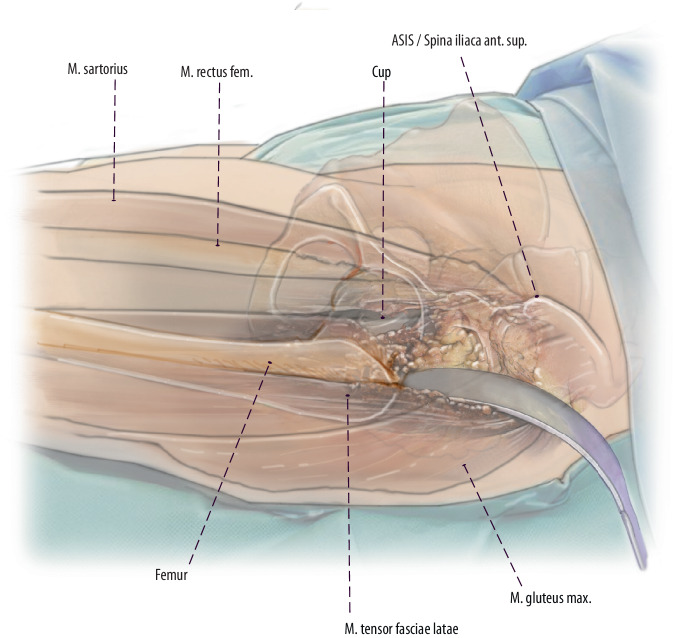
Thus, direct access to the femur is possible after TFL release, which enables the use of long straight modular and non-modular stems and revision (explantation) instruments. We recommend to carry out this release and not to release the external rotators if possible. The tensor can be easily reattached by two or three resorbable sutures. (left hip)

**Fig. 6:**
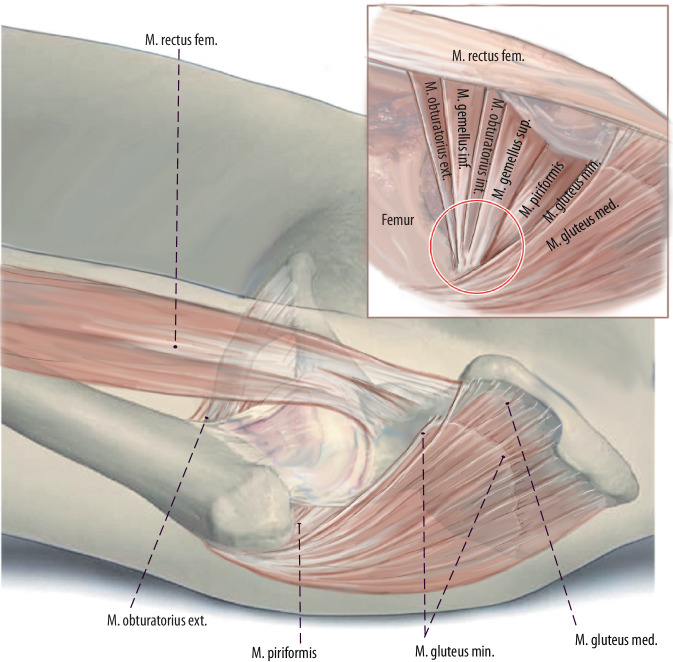
This drawing shows an anterior view to the external rotators of the hip joint. If TFL release still does not produce adequate femoral exposure, the piriformis tendon and/or the conjoined tendon (tendons of gemellus superior muscle, obturator internus muscle, gemellus inferior muscle) is the next to be released, ensuring avoidance of inadvertent damage to the other greater trochanteric tendons and/or abductor musculature, as well as avoiding piriformis and obturator externus tendon releases. Therefore, the dorsal capsule has to be released as well. However, in the authors’ experience this release can be avoided in most femoral revision cases. (left hip)

**Fig. 7:**
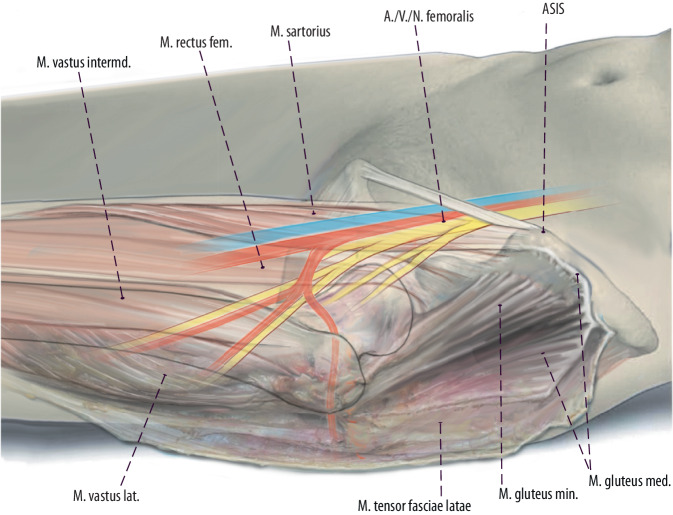
If the femoral diaphysis has to be approached either a second skin incision, a longitudinal extension or a lazy-S extension of the DAA can be performed (Fig. 2). If a longitudinal skin incision is performed, the course of the femoral nerve has to be respected with utmost care. The skin incision is carried down distally from the primary longitudinal incision aiming towards the lateral edge of the patella. One retractor is put at the level of the lesser trochanter. The proximal branch of the femoral nerve runs distally to this retractor in most cases. The proximal branch is identified at a distance approximately 1 fingerbreadth distal to the upper margin of the lesser trochanter. This branch is usually 1 cm wide and runs superficially. The second branch is identified more distally. The internervous plane between the two branches is developed and this interbundle interval is on average 3.3 cm wide. The vastus intermedius muscle is easily identified in this interbundle interval of the femoral nerve and can be easily split. Then blunt retractors are placed submuscularly around the femur between the two bundles. The distal bundle is located at the level of the femoral isthmus. At this level, the femur should be approached underneath the vastus lateralis and intermedius. However, this technique has some limitations and is technically demanding. The interbundle technique is limited to the level of the distal branch of the femoral nerve. The distal branch should be left intact. Therefore, in case the femur needs to be exposed at the level of the distal branch, the entire vastus lateralis and intermedius muscle needs to be lifted off the femur at this level. Minimizing traction at the branch is done by mobilizing the retractors gently during exposure. In muscular patients, it can be more difficult to obtain an optimal view because of the bulky muscular volume. (left hip)

**Fig. 8:**
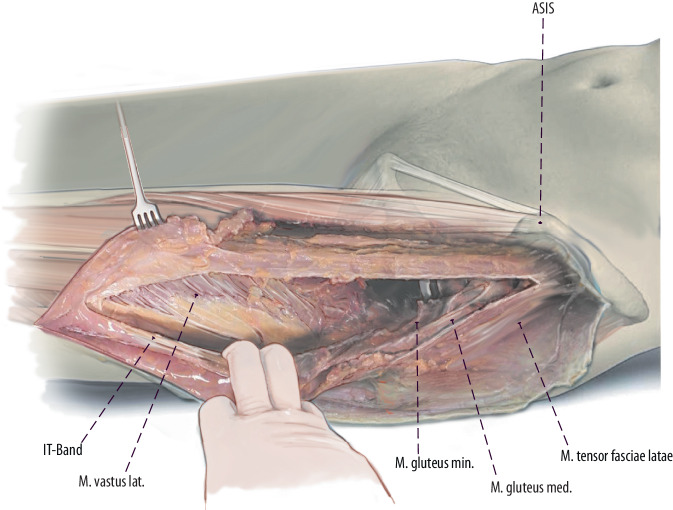
Most DAA revision surgeons prefer to curve the incision laterally from the point most distal to the original DAA approach, forming a “lazy-S”-shaped skin incision (Fig. 2). Then the thin fascia between anterior boarder of the TFL and the quadriceps muscle is split longitudinally as far as distally needed. Then the fascia, the TFL muscle and the iliotibial (*IT*) band can be bluntly mobilized from the underlying vastus lateralis muscle to lateral. If an extended trochanteric osteotomy (ETO) is performed, the vastogluteal muscle sling cannot be fully preserved because the anterior half of the vastus lateralis is split in line with its fibers. (left hip)

**Fig. 9:**
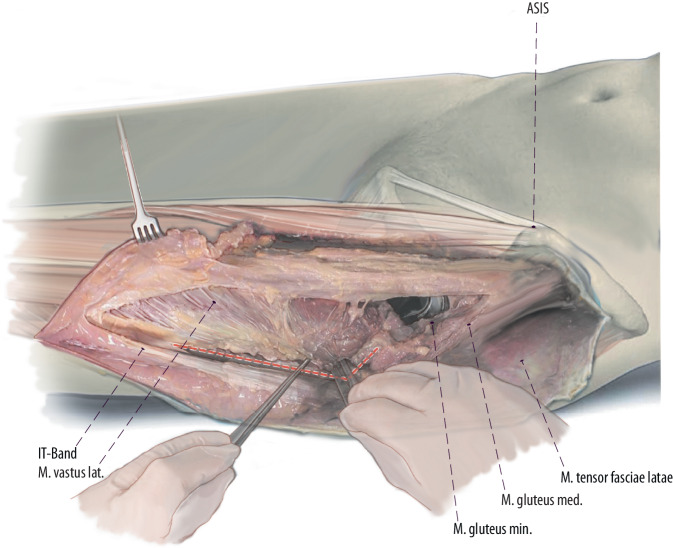
Thus, the posterior border of the vastus can be exposed by pulling the fascia laterally and internally rotating the leg. A subperiosteal dissection of the vastus lateralis muscle from posterior lateral to anterior medial can now be performed. This dissection can either be performed with a Cobb instrument or a cautery. (left hip)

**Fig. 10:**
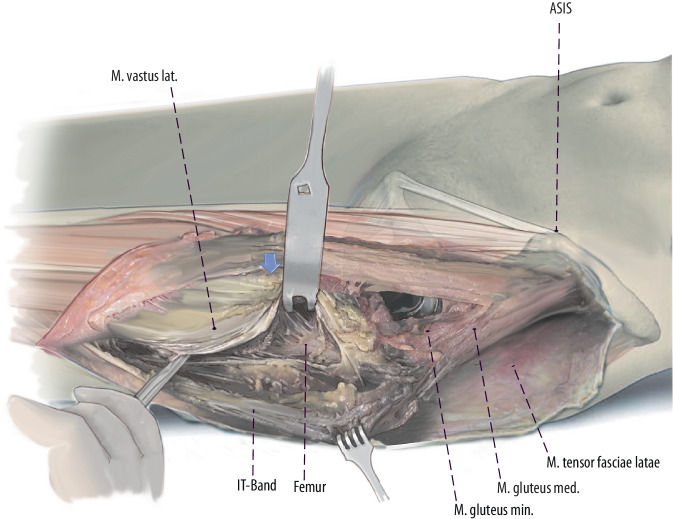
Mobilization of the vastus lateralis is started distally and laterally to the greater trochanter. To ensure a bony blood supply, a muscular attachment at the anterior aspect of the femur should be preserved. Perforating arteries are responsible for the bloody supply of the vastus lateralis muscle. Therefore, this mobilization of the vastus lateralis muscle has to be performed with care to cauterize or ligate the perforating arteries. (left hip)

**Fig. 11:**
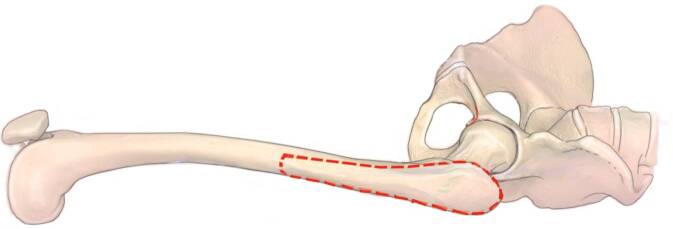
The extension of the interval distally with the lazy‑S extension allows to perform an extended trochanteric osteotomy (ETO) or a transfemoral osteotomy (Wagner) in case of well-fixed femoral components. Other indications are the removal of cement in the femoral diaphysis, treatment of periprosthetic fractures and in difficult primary THA (e.g., proximal femoral deformity, hip dysplasia). The ETO (*red dodded line*) is performed on the lateral aspect of the femur and carried out distally. The aim of this osteotomy is to maintain an intact muscle-osseous sleeve which is composed mainly of the gluteus medius, greater trochanter, vastus lateralis, and femoral diaphysis fragment. (left hip)

**Fig. 12:**
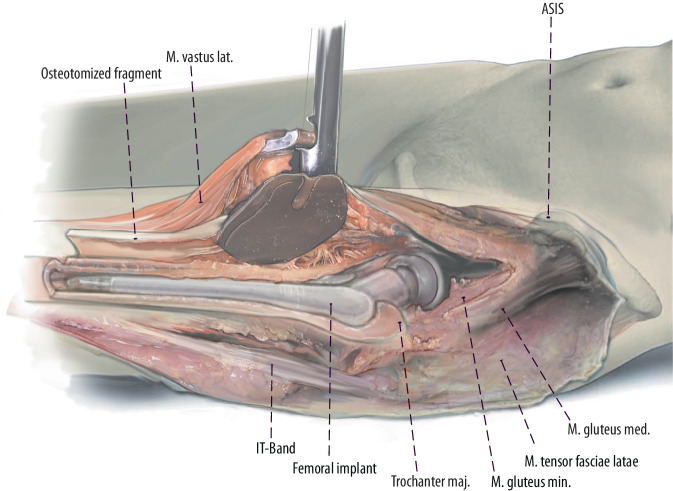
The femoral osteotomy is performed from anterior to open the femur fragment posteriorly. For this step the vastogluteus muscle sling has to be released. Multiple perforations of the bone at the osteotomy site with the use of a drill or high-speed burr in regular intervals might avoid creating a potential stress riser at the junction of the vertical and the horizontal cuts. A saw is used to cut the anterior lateral aspect of the cortical bone, lateral to the femoral component into the femoral canal. Then the osteotomy can be completed posteriorly. A distal transverse cut is completed up to implant with a saw or an osteotome. Then a posterior hinge is created and the osteotomy is elevated carefully and gradually by two osteotomes to avoid a fracture of the osteotomy fragment. (left hip)

**Fig. 13:**
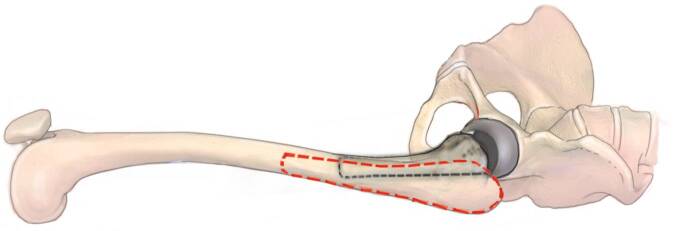
Another excellent option to create a cortical window is the transfemoral approach (Wagner osteotomy). The transfemoral osteotomy (*black dodded line*) facilitates excellent exposure of the femoral canal, while preserving hip abductors and vastus lateralis musculature in continuity. The vastus is thought to counteract the pull of the abductors in the coronal plane, thus, avoiding proximal migration and promoting bony union at the osteotomy. The anterior one-third of the femoral bone should be osteotomized. We recommend again to perforate the femur with a drill in regular intervals to avoid stress risers. Then the drill holes are combined with an oscillating saw, while protecting the soft tissues with two Hohmann retractors. (left hip). (*red dodded line*: ETO)

**Fig. 14:**
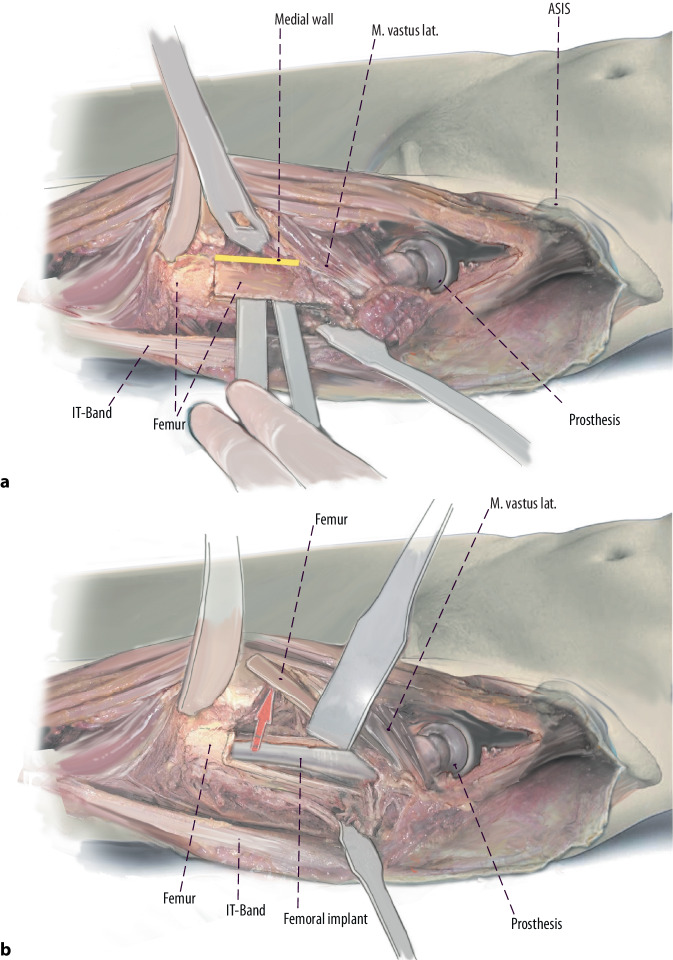
At the end of the horizontal osteotomy, a transverse osteotomy should be performed. Then a saw or an osteotome can be used to cut the medial aspect of the cortical bone (**a**). Finally, the osteotomy can be completed and elevated carefully and gradually by two osteotomes to avoid a fracture of the osteotomy fragment until the femoral stem is exposed (**b**) (left hip)

## Special surgical considerations

(Figs. 15 and 16)

**Fig. 15:**
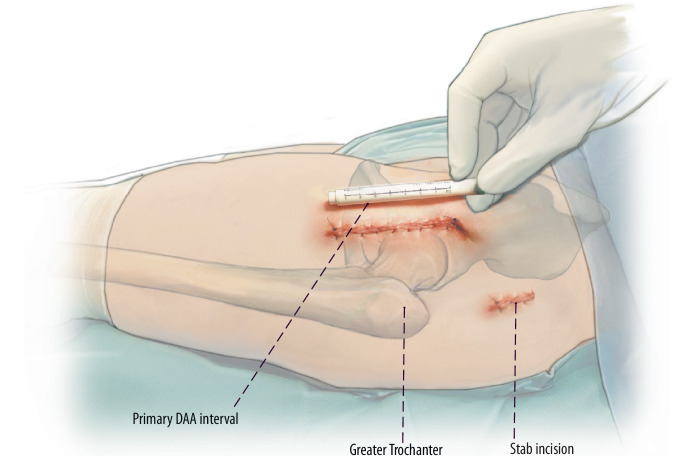
As an alternative method, especially in the learning curve during femoral revision surgery with the DAA a stab incision might be considered. Therefore, the greater trochanter is palpated and the stab incision is made 5–10 cm proximal to the tip of the greater trochanter in line with the axis of the femur. The fascia of the gluteus medius muscle is split bluntly using scissors. Similar to a percutaneous nail insertion, a protection sleeve is used to insert instruments and implants

**Fig. 16:**
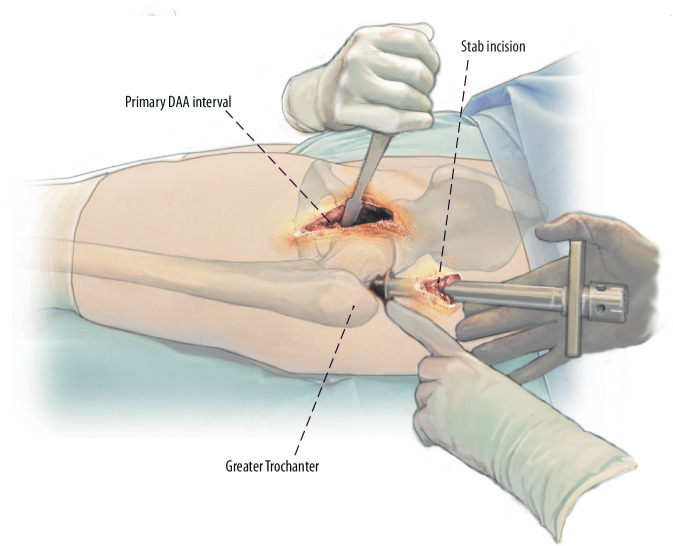
For this purpose, we usually use a 20 mL syringe that is freed from its plunger and cut open at its anterior part. Reamers and trial implants can be introduced through this protection sleeve. After implantation of the stem, the proximal femur has to be reamed to accommodate the modular neck adapter. For this purpose, modular instruments are needed that make it possible to introduce the reamer via the DAA and the connector via the gluteal mini-approach. Accordingly, the modular neck segment of the original prosthesis is implanted via the DAA while the definitive implants are coupled via the gluteal approach. With this technique, there is no need for any extension of the primary DAA interval

## Postoperative management

- Weight bearing and physical therapy protocols depending on the amount of femoral revision
- Anticoagulation therapy: low molecular weight heparin for 35 days after surgery
- Prophylaxis of periprosthetic ossifications for 10 days after surgery with nonsteroidal anti-inflammatory drugs

## Errors, hazards, complications

- ETO (Extended trochanteric osteotomy): non-union rate of 2–7%, failure of an ETO can lead to abductor weakness, severe pain, gait abnormalities and loosening of the femoral component. Therefore, careful and gradual elevation of the osteotomy fragment should be performed to avoid a fracture of the fragment. In symptomatic patients, revision surgery with a cable grip system can be performed.
- Interbundle technique: Sensory nerve deficit of the lateral femoral cutaneous nerve (LFCN). Neuropraxia of the femoral nerve can occur in case too much traction is applied. In such a scenario, short-term follow-up with an electromyogram should be performed 10 days after the surgery. In cases of motoric deficits debridement of the femoral nerve might be considered.
- All approach extensions: numbness of the anterolateral thigh due to LFCN injury. Longitudinal extension of the DAA (Direct Anterior Approach) entails a higher risk of harming the LFCN. Lazy S‑type distal extension: reduced risk of jeopardizing the LFCN.

## Results

A total of 50 femoral revisions with a mean age of 65.7 years (min 50.3; max 83.7) were retrospectively included. The mean follow-up was 2.1 years and the average body mass index was 27.9 (range 18.6–42.2). The previous approach for the primary THA was a direct lateral approach (Hardinge) in 29 cases, a posterior approach in 1 patient, and a DAA in 20 patients.

The femur was approached endofemoral in 41 cases, while a transfemoral approach with a lazy‑S extension was performed in 9 surgeries. No stab incision was needed in any of the cases. An additional cup revision was done in 22 cases (uncemented cup: 10; cemented: 2; reconstruction cage: 10). The mean cut-suture time was 125 min (range 41–250 min). The overall complication rate was 12% (6 complications). Three patients had a dislocation which was treated by closed reduction. Three patients or 6% of the included patients required reoperations. One patient suffered from a periprosthetic joint infection and was treated with a two-stage revision. One patient required cup revision because of recurrent postoperative dislocations. One patient had a postoperative fall resulting in a periprosthetic fracture which was again treated with a stem revision and cerclage wires. The average time to revision was 6 months (range 12 weeks to 23 months). During the follow-up period no subsidence or signs of radiolucency were found. Two patients had heterotopic ossification at the final follow-up investigations, which did not require any revision surgery. There had been no cases of mechanical loosening or stem fracture. Median total WOMAC (Western Ontario and McMaster Universities Osteoarthritis Index) score before surgery was 52.5 (interquartile range [IQR]: 33.3) and improved to 27.2 (IQR: 30) after surgery. Mann–Whitney test demonstrated a significant difference for all subcategories between preoperative and postoperative.

### Interbundle technique (longitudinal extension).

In another consecutive series of 6 patients undergoing the interbundle technique, electromyography (EMG) was used to evaluate the integrity of the femoral nerve. In 5 of 6 patients, EMG findings were normal. In 1 patient, the middle bundle showed neuropraxia. This patient sustained a long periprosthetic fracture extending distally to the middle bundle.

## Declarations

For this article no studies with human participants or animals were performed by any of the authors. All studies mentioned were in accordance with the ethical standards indicated in each case.
